# Direct observation of the CVD growth of monolayer MoS_2_ using in situ optical spectroscopy

**DOI:** 10.3762/bjnano.10.57

**Published:** 2019-02-26

**Authors:** Claudia Beatriz López-Posadas, Yaxu Wei, Wanfu Shen, Daniel Kahr, Michael Hohage, Lidong Sun

**Affiliations:** 1 Institute of Experimental Physics, Johannes Kepler University Linz, A-4040 Linz, Austria; 2 State Key Laboratory of Precision Measuring Technology and Instruments, Tianjin University, Weijin Road 92, Nankai District, 300072 Tianjin, China; 3 Nanchang Institute for Microtechnology of Tianjin University, Weijin Road 92, Nankai District, 300072 Tianjin, China

**Keywords:** chemical vapor deposition (CVD), in situ differential optical spectroscopy, molybdenum disulfide (MoS_2_) monolayer, two-dimensional transition-metal dichalcogenides (2D TMDC)

## Abstract

Real-time monitoring is essential for understanding and precisely controlling of growth of two-dimensional transition metal dichalcogenide (2D TMDC) materials. However, it is very challenging to carry out such studies during chemical vapor deposition (CVD). Here, we report the first, real time, in situ study of the CVD growth of 2D TMDCs. More specifically, the CVD growth of a molybdenum disulfide (MoS_2_) monolayer on sapphire substrates has been monitored in situ using differential transmittance spectroscopy (DTS). The growth of the MoS_2_ monolayer can be precisely followed by observation of the evolution of the characteristic optical features. Consequently, a strong correlation between the growth rate of the MoS_2_ monolayer and the temperature distribution in the CVD reactor has been revealed. Our results demonstrate the great potential of real time, in situ optical spectroscopy to assist the precisely controlled growth of 2D semiconductor materials.

## Introduction

Two-dimensional transition metal dichalcogenide (2D TMDC) materials have drawn wide attention because of their fascinating physical and chemical properties [1–6]. Given that the potential advantages of 2D TMDCs used as the active materials for various devices have been established, the primary focus is now the cost-efficient, reliable, and high-throughput synthesis of 2D TMDCs using processes compatible with current semiconductor technology. Various approaches for synthesizing large-area 2D TMDCs have been reported, including mechanical exfoliation, sulphurization of metal thin films, mass transport, molecular beam epitaxy (MBE) and chemical vapor deposition (CVD) [6–7]. In particular, CVD is considered to be an attractive and very promising approach for large-scale synthesis of 2D TMDCs [8–14]. As a result, numerous empirical attempts have been taken to optimize the CVD process by choosing appropriate precursors, supporting substrates, carrier gases, flow rates, synthesis temperature, etc. Although these studies have created helpful growth recipes and have inspired many theoretical discussions about the growth mechanisms, they were still unable to provide a clear path to the full understanding of the growth mechanisms [10,15–19]. Several recent efforts aiming at manipulating the nucleation and growth of 2D TMDCs have successfully led to the great improvement of the film quality and a well-orientated MoS_2_ monolayer has even been fabricated in wafer scale [12,14,16,19]. These results highlight the important role of surface kinetics in the CVD growth of the 2D TMDCs. In order to improve our understanding of the surface kinetics involved, in situ studies performed during the CVD growth of 2D TMDCs are essential. However, to the best of our knowledge, no study in this direction has been reported despite its importance. Consequently, the growth mechanisms are still the subject of much speculation.

Because typical CVD processes used for the synthesis of 2D TMDCs are very complicated, involving gases with pressure in the range between atmosphere and several mbar, the characterization methods based on electron beam techniques are not applicable and the in situ real time study becomes very challenging. On the other hand, each type of 2D TMDC material possesses specific optical properties which are characteristic of their chemical composition, crystalline structure, and number of layers [2,20]. Their optical properties are also sensitive to extrinsic modifications, for instance, interaction with substrate [21], gas molecule adsorption [22–23] as well as strain field [24]. These facts make optical spectroscopy a sensitive probe to monitor the surface kinetics involved during the growth of 2D TMDCs. Furthermore, the optical methods used in the visible range can be applied under various environments including vacuum, atmosphere, and even high pressure conditions. Consequently, optical spectroscopy is the method of choice for in situ studies during CVD growth. Monitoring the evolution of the optical properties of 2D TMDCs during CVD growth may provide a sensitive means to assess the surface processes and to characterize the morphology and crystalline structure of the films. By systematic study using in situ optical spectroscopy assisted with other ex situ characterization techniques, the details of the kinetics including adsorption, dissociation, reaction, nucleation, and growth can be revealed.

Differential reflectance spectroscopy (DRS), which measures the normalized difference between the reflectance of the bare surface and the surface covered by thin films, possesses an enhanced sensitivity to the surface modification and ultrathin film growth [25–26]. This technique has been successfully applied to reveal the optical properties of 2D TMDCs [2,20] and, most recently, also to monitor the molecular beam epitaxy of monolayer MoSe_2_ on sapphire substrates [27]. In the current work, we have applied an analogous technology, namely, differential transmittance spectroscopy (DTS), to realize the in situ real time study of the CVD growth of monolayer MoS_2_ on Al_2_O_3_(0001) surface. In this case, the normalized difference between the transmittance through the bare substrate and the transmittance after a given deposition time are resolved. By monitoring using DTS during growth, the evolution of the optical properties associated with the MoS_2_ layer can be revealed spectroscopically. Furthermore, since the sapphire substrate is transparent in the visible range, the change of the reflectance upon the growth of an ultrathin MoS_2_ layer is directly proportional to its absorption [25]. Assuming the scattering effect is negligible, for the van der Waals epitaxy of MoS_2_ on the transparent sapphire substrate, the DT spectrum can thus be generally associated with the absorption of the adlayer. Consequently, the growth can be monitored in situ and in real time, and the detailed information associated with the kinetics can be deduced from the DT spectra.

## Results and Discussions

### Ex situ characterization

**Figure 1:**
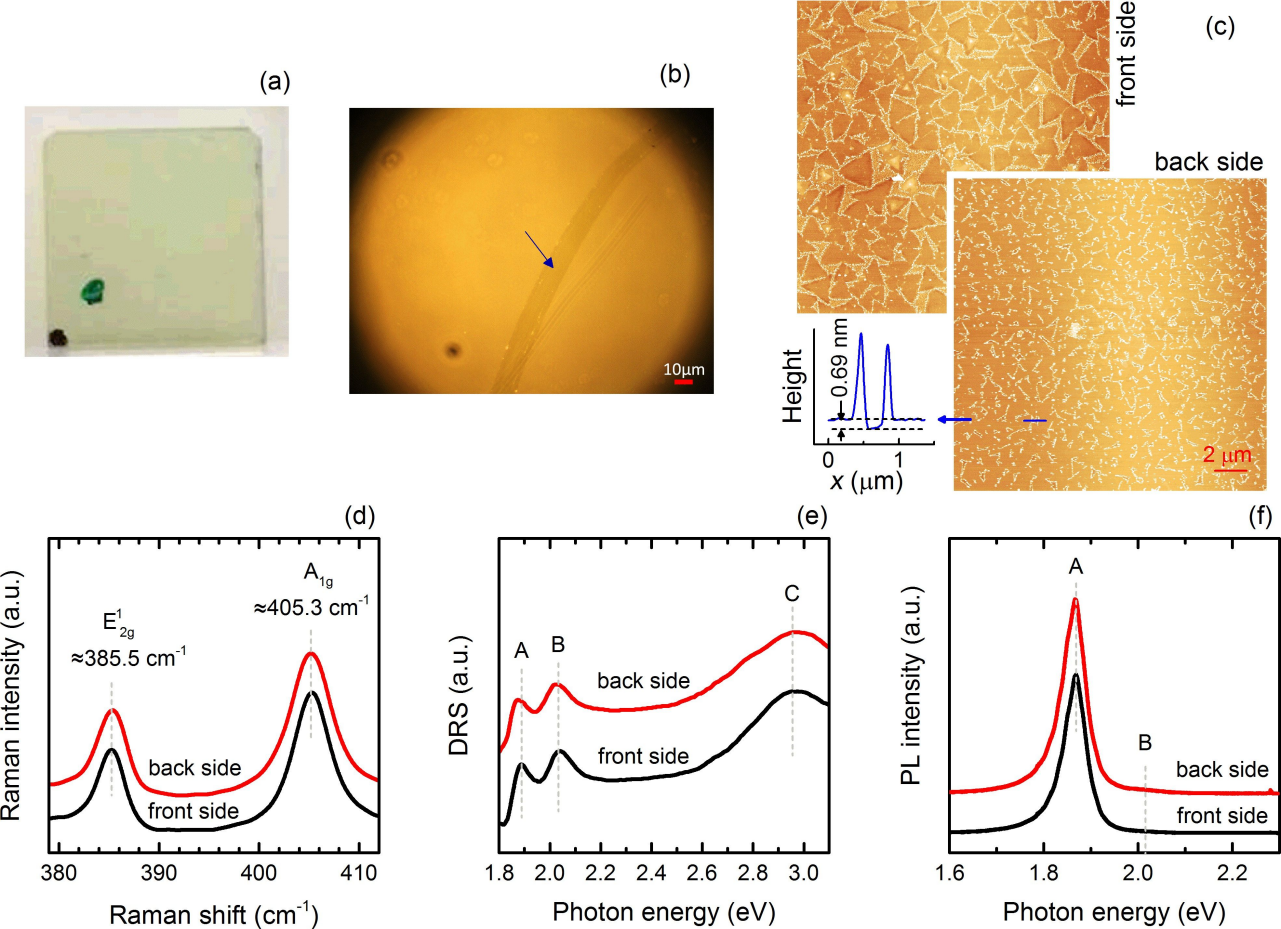
The results of the ex situ characterization. (a) A photograph representing the 10 × 10 mm substrate after CVD growth. The black and green dots marked on the front and back sides of the substrate, respectively, are used to distinguish between the two surfaces. (b) Optical microscopy image taken from the surface at the front side of the same substrate. A scratch is indicated by the arrow. (c) AFM images recorded from the surfaces at the front and back side of the substrate, respectively. The height profile is measured along the blue solid line in the image. (d), (e) and (f) The Raman, differential reflectance, and photoluminescence spectra recorded on the surfaces at the front and back side of the substrate, respectively.

Following the CVD process described in the Experimental section, an MoS_2_ monolayer was deposited, simultaneously, on not only the surface on the front side (face to the Ar flow direction) but also on the back side of the sapphire substrate. This conclusion is based on a thorough ex situ characterization after CVD growth using differential reflectance spectroscopy (DRS), Raman spectroscopy, photoluminescent spectroscopy (PL), optical microscopy (OM), and atomic force microscopy (AFM). Actually, from the first glance at the substrate after CVD (see Figure 1a), it can be recognized that the substrate shows a homogeneous light green colour, which is characteristic for a sapphire substrate covered by an MoS_2_ monolayer. Indeed, OM images (Figure 1b) taken in different areas of the front and back sides of the substrate reveal that the surfaces at both sides are covered by homogeneous layers, which can only be recognized by the appearance of defects. Figure 1c presents the AFM images measured at the front and back sides of the sapphire substrate confirming that both surfaces are covered by an almost complete monolayer of MoS_2_. On the front surface, incompletely merged triangular-shaped MoS_2_ single crystals are observed, along with large nanoparticles at the edges of the crystalline MoS_2_ and small ones distributed elsewhere on the surface. The lateral size of the crystalline MoS_2_ grains is in the order of micrometers. In contrast, the large nanoparticles at the edges of MoS_2_ single crystals exhibit diameters of approximately 50 nm and heights around 10 nm, whereas the small ones show diameters around 20 nm and heights around 1 nm (see Supporting Information File 1, Figure S1). Similar morphology has been observed previously on the CVD grown 2D TMDCs samples. Most recently, taking the CVD growth of MoSe_2_ as an example, Li et al. have demonstrated that the small nanoparticles are associated with an intermediate reactant for MoSe_2_ growth, whereas the large nanoparticles are byproducts of the MoSe_2_ [28]. Due to the fact that the areas of the surface between the triangular-shaped MoS_2_ crystals are fully covered by these nanoparticles, no reasonably large surface areas of bare substrate can be found. Consequently, the thickness of the triangular-shaped MoS_2_ crystals cannot be determined from the AFM height profile measurement. However, the corresponding Raman and PL spectra presented in Figure 1d and Figure 1f indicate that these triangular-shaped MoS_2_ crystals should be monolayer thick. The AFM images taken from the back surface of the substrate show almost completely merged MoS_2_ grains together with large nanoparticles at the grain boundaries. Furthermore, the height profile measured across the edge of the MoS_2_ layer reveals a layer thickness of ≈0.69 nm, which is very close to the interlayer spacing in the bulk of 2H phase MoS_2_. The Raman spectra recorded from each side of the substrate (Figure 1d) show the characteristic peaks at 385.5 and 405.3 cm^−1^, which are attributed to the in-plane (E^1^_2g_) and out-of-plane (A_1g_) vibration modes of the 2H MoS_2_ crystal. Most importantly, the interval between these two peaks is ≈ 20 cm^−1^, which is characteristic for the MoS_2_ monolayer [29]. The DR spectra measured at the surfaces on the front and back sides of the substrate are plotted in Figure 1e. The peaks marked by A and B, which are located around 1.88 and 2.03 eV, are due to the excitonic transitions occurring at the K and K^′^ points of the Brillouin zone, respectively. The broad peak C around 2.95 eV is attributed to the interband transitions transpiring near the critical point of Γ, where the valance and conduction bands are nested [30]. The relaxation of the transitions A and B gives strong PL emission, which is plotted in Figure 1f. The strong PL emission corroborates the monolayer thickness of the MoS_2_ layer. In order to check the homogeneity, all the measurements have been performed at several different spots over the substrate surfaces and the results are rather identical. Consequently, a homogeneous MoS_2_ monolayer has been synthesized on both the front and back sides of the substrate. However, a closer inspection reveals some detailed differences between the morphology at the front and back surfaces regarding the surface coverage and the grain size. The grains grown on the back surface have already coalesced, forming a rather compact monolayer. In contrast, the grains on the front surface are still rather separated. Furthermore, it appears that grain size is relatively large on the front surface. These observations indicate a difference between the front and back side of the substrate regarding the growth kinetics. We attribute the observed distinction tentatively to the possible deviation of the effective deposition rate on each side of the substrate. Actually, the deposition rate could be enhanced on the back surface due to the interruption of the gas flow by the substrate, which may introduce a local turbulence and in turn a higher deposition rate.

### In situ real time measurement

**Figure 2:**
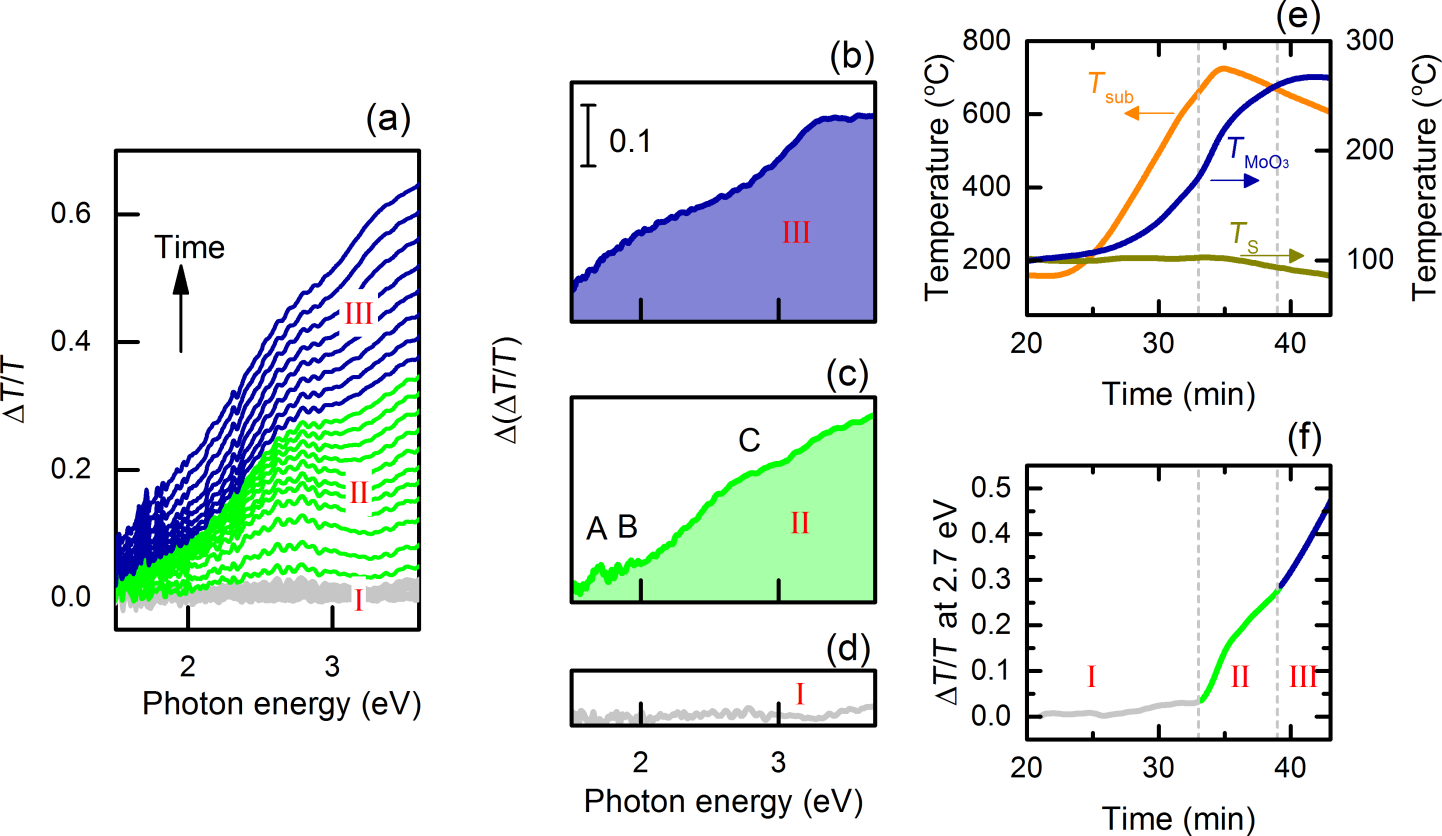
(a) Differential transmittance (DT) spectra (Δ*T*/*T*) recorded during CVD growth. The time interval between the adjacent spectra is 30 s. (b), (c) and (d) The incremental changes of the DT spectrum over the third, second and first sections of the growth, respectively. (e) The temperature profiles of the substrate (*T*_sub_), MoO_3_ source (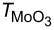) and the sulfur source (*T*_S_) recorded during growth. (f) The corresponding DT intensity at 2.7 eV recorded during CVD growth.

The evolution of the optical properties during growth can be recognized by a close look at Figure 2a, which shows the DT spectra recorded during the CVD process. Based on the evolution of the DTS, we divide our CVD process into three sections. For the first section (section I) starting from the preheating of the sulfur source and the furnace, it can be seen that the DT spectrum remains unchanged during the first 33 min, although the temperature measured at the position related of sulfur, MoO_3_, and the substrate increased. This observation shows that no thin film was deposited during this period. The second section (section II) starting from the time *t* = 33 min, transmittance around 2.7 eV decreases (DT signal increases) with time. The energy position of the DTS feature at 2.7 eV coincides very well with the absorption peak C of the MoS_2_ monolayer. The continuous decrease of the optical intensity at this energy indicates the increase of the optical absorption due to the growth of the MoS_2_ layer on the surfaces of the sapphire substrate. In comparison with the DR spectra of the same sample measured ex situ at room temperature (see Figure 1e), the peak C is broad and shifted to lower energy. Furthermore, the sharp absorption features A and B observed at room temperature are hardly visible. We attribute this deviation from the optical spectrum measured at room temperature to the high substrate temperature (≈700 °C) during growth. Indeed, the study of the temperature dependence of the absorption of the MoS_2_ monolayer shows the same tendency [31]. Therefore, the DT spectra recorded after more than 33 min of deposition show characteristic absorption of the MoS_2_ monolayer at elevated temperature where the amplitudes increase with the deposition time. However, during the third section (section III), which starts at about 39 min, the DT signal at a higher energy of 3.2 eV begins to dominate. Based on its energy position, this feature can be attributed to the absorption of MoO_3_ [32–33]. As a semiconductor, MoO_3_ shows a bandgap around 3 eV. Considering the high temperature of the substrate, we can exclude the possibility that the MoO_3_ thin films were formed on the substrate surfaces. Actually, we attribute the observed DTS signal to the absorption of the MoO_3_ thin films which were deposited on the inner surface of the quartz window at the end of the furnace tube. Since the quartz was maintained close to room temperature, it is reasonable to assume that a small amount of the MoO_3_ molecules carried by the Ar flow from upstream may be deposited on the inner surface of this quartz window. The deposition rate of the MoO_3_ on the inner surface of the quartz window should be proportional to the product of 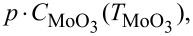 in which, 
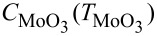
 is the concentration of MoO_3_ molecule above the MoO_3_ source which depends exponentially on the source temperature 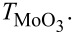 The coefficient *p* accounts for the transportation efficiency of MoO_3_ molecules from the source to the inner surface of the quartz window. Although the coefficient *p* has been minimized, it was not, however, insignificant in this study. Actually, the growth of the MoO_3_ feature begins already in the second section, where the DT signal increase at 2.7 eV was accompanied by the small increase at 3.2 eV.

The characteristic increments of the DT spectrum during each section can be recognized from the Δ(Δ*T*/*T*) spectra plotted in the central column of Figure 2. Indeed, the increment of the DT signal over the large wavelength range is negligible during the section I (Figure 2d) indicating no growth occurring on the substrate surfaces. For the section II (Figure 2c), the increase of the DT signals can be clearly recognized not only at 2.7 eV but also at 1.85 and 1.72 eV associated with the absorption peaks C, B and A of the growing MoS_2_ monolayer, respectively. In comparison with the DR spectra measured ex situ at room temperature, a systematic red shift can be observed for all the three features verifying the influence of the substrate temperature. In addition, the absorption of the MoO_3_ thin films deposited on the quartz window is also clearly visible at 3.2 eV. Finally, during the section III (Figure 2b), the incremental change of the DTS is dominated by the increase in the energy range above 3.2 eV. In contrast, the features associated with MoS_2_ vanish. The decrease of the transmittance (the increase of DTS) over the low-energy range could be attributed to the increase of the scattering of the MoO_3_ thin film deposited on the quartz window.

In order to understand the observed evolution of the DT spectroscopy, its correlation with the temperature at the positions related to the sulfur source, MoO_3_ source, and the substrate are plotted in Figure 2e and Figure 2f. In Figure 2f, the DT signal at 2.7 eV is selected to represent the growth of the MoS_2_. By looking at Figure 2e and Figure 2f, it becomes clear that the growth of the DT signal at 2.7 eV and thus the MoS_2_ layer sets in when the temperature of the substrate and MoO_3_ exceed 650 °C and 170 °C, respectively. This is based on the following two facts: (1) The sulfur source had been being maintained at a temperature above 100 °C for more than 10 min until this moment. (2) The growth of the MoS_2_ monolayer at a substrate temperature as low as 530 °C has been reported [11]. We thus attribute the observed onset of the MoS_2_ growth to the evaporation of the MoO_3_ reaching a recognizable rate. Afterwards, the DT signal at 2.7 eV increases monotonically revealing the ongoing growth of the MoS_2_ layer. Interestingly, although the temperature of the MoO_3_ source was still increasing, the growth speed of the DT signal at 2.7 eV dropped at around 35 min. With a closer inspection of the temperature curves, the observed decrease of the growth rate at 2.7 eV can be correlated to the declining substrate temperature and the sulfur source temperature. Actually, without going into the details of the kinetics of the reaction, the growth rate *r* of the MoS_2_ can be associated with several factors that depend strongly on the temperature of the sulfur source (*T*_S_), MoO_3_ source (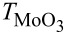) and substrate (*T*_sub_), respectively, in the following way:

[1] $$r\propto k\left(T_{\text{sub}}\right)\cdot C_{{\text{MoO}}_{3}}\left(T_{{\text{MoO}}_{3}}\right)\cdot C_{\text{S}}\left(T_{\text{S}}\right)\;,$$

where *k*(*T*_sub_) represents the effective reaction rate coefficient between MoO_3_ and sulfur leading to the formation of MoS_2_. The *k*(*T*_sub_) depends strongly on the *T*_sub_ as indicated by the Arrhenius equation. Similar to 
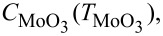
*C*_S_(*T*_S_) is the concentration of the sulfur molecules over the sulfur source, which depends exponentially on *T*_S_. This relation emphasizes the strong influence of the temperature on the growth rate of MoS_2_. Indeed, the fast rise of the DT signal at 2.7 eV at the initial stage of the section II can be correlated to the increase of the temperature at both the MoO_3_ source (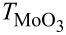) and around the substrate (*T*_sub_). The decrease in the growth rate of the DT signal at 2.7 eV observed at ≈35 min coincides with the decrease of the temperature around the substrate (*T*_sub_) and at the source of sulfur (*T*_S_). The recovery of the growth rate of DT signal at 2.7 eV within section III cannot be attributed to the absorption of MoS_2_ anymore. Instead, it is induced by the overall decrease of transmittance through the quartz window due to the enhanced coating of the MoO_3_ layer. Indeed, in contrast to the decrease of *T*_sub_ and *T*_S_, the MoO_3_ source temperature 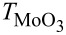 continues to increase even at the beginning of section III (see Figure 2e).

## Conclusion

In conclusion, we have successfully grown MoS_2_ monolayers on both sides of the c-plane of double polished sapphire substrates using CVD. The monolayers on both sides of the substrate were homogeneously distributed over the 10 × 10 mm substrate surfaces. Most importantly, the evolution of the optical transmittance of the substrate has been monitored in situ in real time during the CVD growth using DTS. The formation of the MoS_2_ monolayers is clearly visible from the development of the DT spectrum. More specifically, the onset of the growth and the variation of the growth rate of MoS_2_ monolayers can be determined. This detailed information about the growth deduced from the DTS results can be correlated very well to the variation of the temperature of the substrate, the MoO_3_, and the sulfur sources. The current results emphasize the importance of the in situ real time study and pave the way for deeper understanding, and eventually, the precise controlled growth of 2D semiconducting materials.

## Experimental

**Figure 3:**
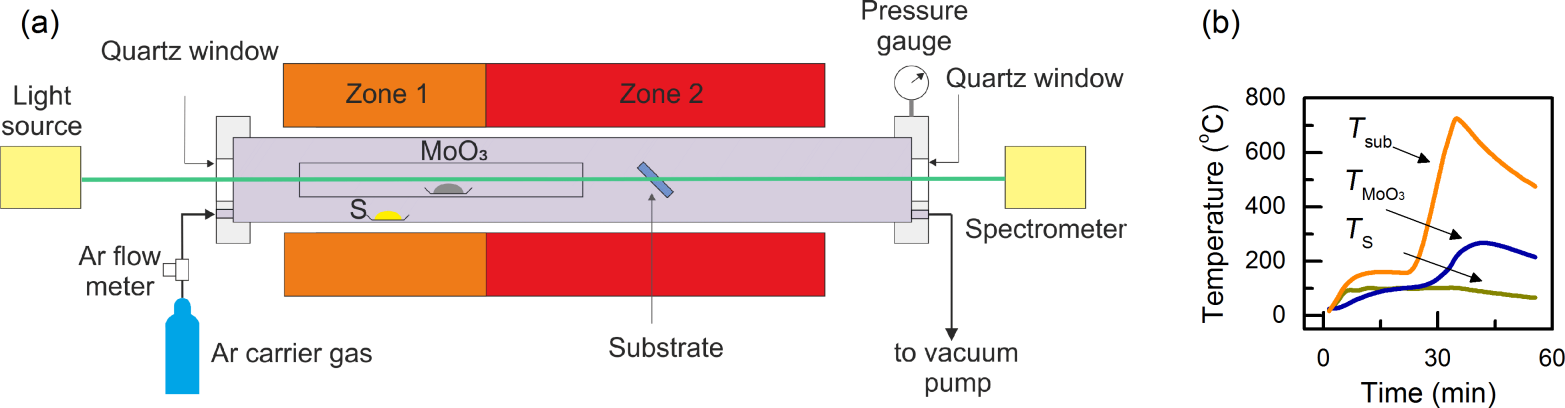
(a) Schematic for the CVD growth of monolayer MoS_2_. (b) Temperature profiles for the substrate, MoO_3_ and sulfur.

The setup of the CVD reactor including the optical spectrometer applied for the in situ measurement is sketched in Figure 3a. High purity MoO_3_ powder is used as the precursor for molybdenum, whereas the sulfur is supplied by vaporizing solid sulfur. High purity Ar was used as a carrier gas and a flow rate of 150 sccm was maintained during the whole process. The air in the reactor was evacuated before filling with Ar gas and the pressure of Ar was maintained at 0.1 Torr until the end of the process. An horizontal tube furnace with a single heating zone and a heating belt were applied as heating elements for the substrate, MoO_3_ and sulfur, respectively. To establish the temperature distribution required for the CVD, the substrates were positioned at the center of the furnace, while the alumina boats containing solid sulfur and MoO_3_ were located 15 cm and 3 cm, respectively, away from the entrance of the furnace upstream of the Ar flow. From the configuration exhibited in Figure 3a, it can be recognized that the sulfur is predominantly heated by the heating belt, whereas the MoO_3_ powder was heated not only by the heating belt but also by the heater in the furnace in a radiative manner. In order to avoid the early mixing and reaction between MoO_3_ and sulfur, the alumina boat of MoO_3_ was enclosed in a quartz tube suspended coaxially with the outer quartz tube of the CVD reactor. The distance between the exit of the inner quartz tube and the substrate was maintained at 3 cm. The evolution of temperature of the substrate and the boats containing MoO_3_ and sulfur during growth are plotted in Figure 3b. For the growth reported in this paper, double-side-polished c-plane sapphire (Al_2_O_3_(0001)) was selected as the substrate. During deposition, the substrates were inclined with their surface normal 45 degrees off the axis of the quartz tube of the furnace (see Figure 3a). At both ends of the furnace quartz tube, windows made of quartz were mounted to seal the reactor. The optical transmittance of the substrate was measured in real time using a spectroscopic ellipsometer from J.A. Woollam Co. More specifically, a beam of white light generated from a Xe lamp was guided through the substrate along the axis of the CVD reactor and detected by a spectrometer equipped with a CCD detector array. In order to enhance the sensitivity, the differential transmittance spectrum (DTS) at time *t* was calculated using the following equation (Equation 2):

[2] $$\frac{\Delta T}{T}\left(t\right)=\frac{T_{0}-T_{t}}{T_{0}},$$

where *T*_0_ and *T_t_* denote the transmittance spectra of the bare substrate before the CVD process and after time *t*, respectively. The obtained DTS signal 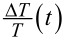 represents the change of the optical transmittance relative to the bare substrate surfaces as a function of time *t*. Because the sapphire substrate is transparent in the wavelength range studied here, the DT spectra measured are directly associated with the absorption of the thin films deposited on the substrate. This fact makes DTS a sensitive method for in situ monitoring of the growth of MoS_2_ monolayers in real time in the current study.

The morphology of the MoS_2_ thin films was investigated ex situ using atomic force microscopy (Veeco Dimensions S3100) in tapping mode. A soft cantilever (TipsNano) was employed. The Raman and PL spectra spectra of the MoS_2_ thin films were collected using a JY Horiba LabRAM Aramis VIS microscope with an excitation wavelength of 532 nm. Measurements were performed in a confocal microconfiguration using a ×100 microscope objective lens and a 2400 grooves mm^−1^ grating. For DR measurements, an inverted microscope (Nikon Eclipse Ti) was used. A beam from a tungsten halogen white light source was reflected into a ×50 ultra-long working distance objective using a neutral density, achromatic beam splitter and then focused onto the sample surface. The reflected signals from the MoS_2_ covered areas (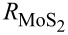) and the bare substrate areas (*R*_substrate_) were collected by the objective separately and sent back through the beam splitter into the spectrometer. The DRS signals were then calculated by 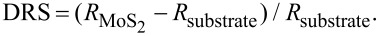 All ex situ measurements were performed at room temperature.

## Supporting Information

File 1 Additional experimental data.
