# Supplementary material for: Direct observation of the CVD growth of monolayer MoS2 using in situ optical spectroscopy
Source: Beilstein J Nanotechnol. 2019 Feb 26;10:557–64. doi: 10.3762/bjnano.10.57 (PMC6404415; doi:10.3762/bjnano.10.57)
Supplement: File 1 — Additional experimental data. [file Beilstein_J_Nanotechnol-10-557-s001.pdf]

BEILSTEIN JOURNAL OF NANOTECHNOLOGY

Supporting Information

for

Direct observation of the CVD growth of monolayer MoS_2 using in situ optical spectroscopy

Claudia Beatriz López-Posadas, Yaxu Wei, Wanfu Shen, Daniel Kahr, Michael Hohage and Lidong Sun

Beilstein J. Nanotechnol. **2019,** *10,* 557–564. doi:10.3762/bjnano.10.57

Additional experimental data

License and Terms: This is a supporting information file under the terms of the Creative Commons Attribution License (http://creativecommons.org/licenses/by/4.0). Please note that the reuse, redistribution and reproduction in particular requires that the authors and source are credited.
The license is subject to the *Beilstein Journal of Nanotechnology* terms and conditions: (https://www.beilstein-journals.org/bjnano)

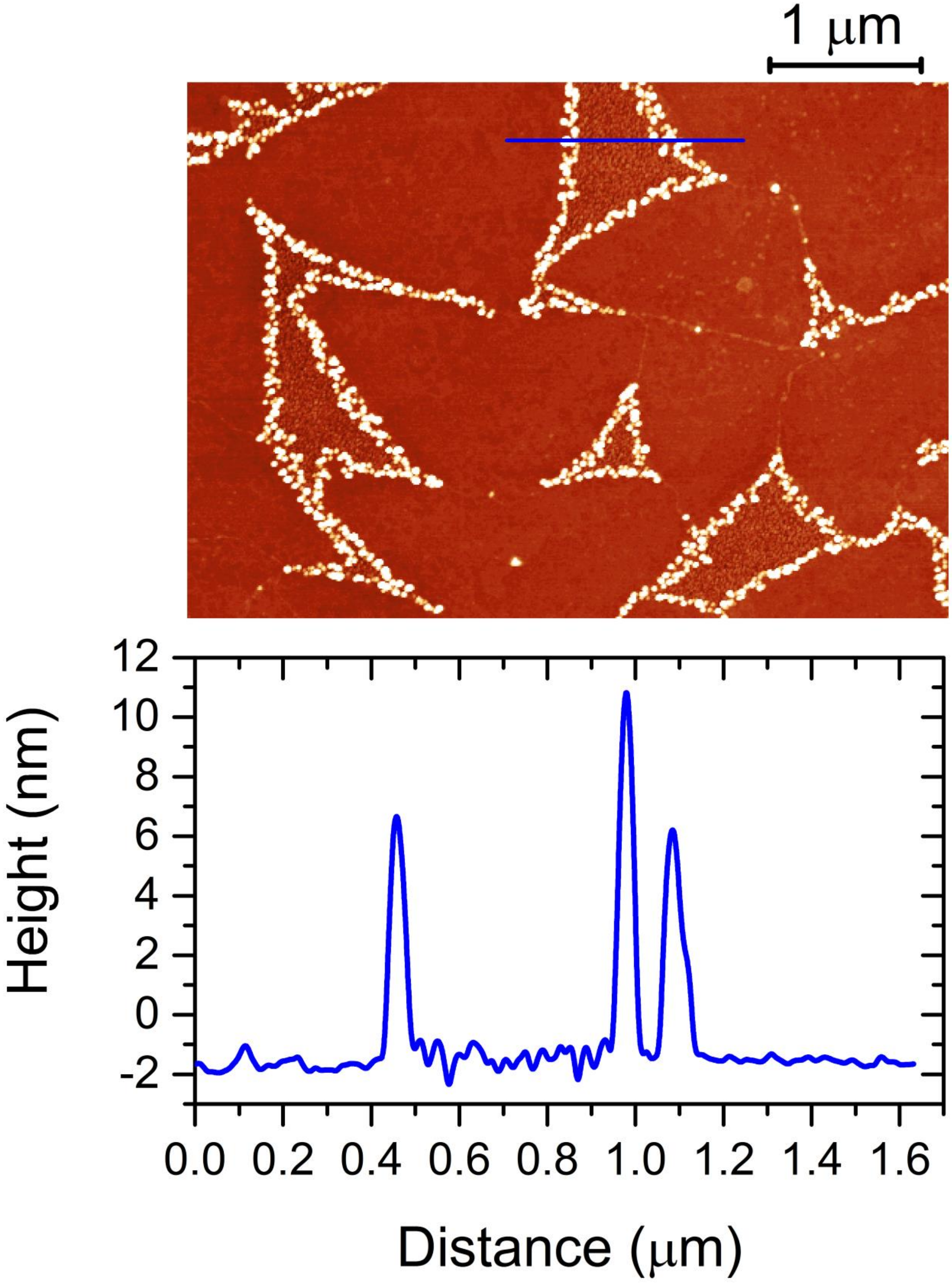

Figure S1: AFM image with a height profile measured from the front surface of the substrate.
